# Design, Synthesis, and Anticancer Activity Evaluation of Hybrids of Azoles and Barbituric Acids

**DOI:** 10.22037/ijpr.2020.113547.14363

**Published:** 2021

**Authors:** Hong-Juan Liu, Xing Huang, Qing-Kun Shen, Hao Deng, Zhiyong Li, Zhe-Shan Quan

**Affiliations:** *Key Laboratory of Natural Medicines of the Changbai Mountain, Affifiliated Ministry of Education, College of Pharmacy, Yanbian University, Yanji, Jilin, 133002, China.*

**Keywords:** Barbituric acid, 1, 2, 3-triazoles, Anticancer, MTT assay, Cell apoptosis

## Abstract

In order to find new drugs with potent antiproliferative effect, a series of novel barbituric acid derivatives containing azoles at the C-5 position were designed, synthesized, and evaluated for antiproliferative activity against three human cancer cell lines (BEL-7402, MCF-7, and HCT-116) using MTT assay. Several of the synthesized compounds exhibited potent antiproliferative effects. The most promising compound was 5-((1-(4-(trifluoromethyl)phenyl)-1*H*-1,2,3-triazol-4-yl) methylene)pyrimidine-2,4,6(1*H*,3*H*,5*H*)-trione (3s), which showed considerably high antiproliferative activity in the BEL-7402 cell line, with a half-maximal inhibitory concentration of 4.02 µM and 20.45-fold higher selectivity for BEL-7402 cells than for normal L02 cells. The apoptosis experiment showed that compound 3s induced apoptosis and cell necrosis in a concentration-dependent manner and exert its anti-proliferative activity. Therefore, compound 3s exhibited better therapeutic activity and specificity compared with the positive control 5-fluorouracil.

## Introduction

Currently, cancer is the second major cause of human death after cardiovascular disease (1). In 2012 alone, 14 million cancer cases and 8.2 million cancer-related deaths have occurred (2). Per the latest information available, it is estimated that if the spread of cancer continues at its present rate, it may cause over 13.1 million deaths in 2030 worldwide (3). Because of its severity, cancer is considered one of the greatest social and economic concerns for the public healthcare system (4). In the last few decades, various approaches for the treatment of cancer have been developed, among which chemotherapy is one of the most fundamental and widely used methods (5).

The lack of effective chemotherapy for cancer is continuously inciting the scientific community to explore new chemical entities for an effective and safe cure for cancer. Therefore, identifying new anticancer agents with higher potency and lower toxicity is a great challenge (6).

Heterocyclic rings represent a molecular framework that serves as a platform for developing pharmaceutical agents for various applications. The antitumor activities of many compounds containing heterocyclic rings have been reviewed (7). For example, the triazole fragment is widely applied in organic, medicinal, and material sciences (8, 9). It has been reported to have a variety of pharmacological effects, including anticancer (10, 11), anti-inflammatory (12, 13), anti-malarial (14), anticonvulsant (15-17), and antidepressant (18-20) activities. Active compounds containing triazole hydrogen bonds and dipole-dipole interactions cause strong dipole moments; thus, they are very durable when hydrolyzed and remain stable under oxidizing and reducing conditions (21). The modiﬁcation strategy of natural products such as compounds **B**, **C**, **D** has attracted more attention. In the preliminary research of this research group, we found some successful cases of the natural product hybrid triazole fragments with anti-tumor activity. Compound **B** is a synthetic xanthotoxin derivative designed, which exhibited antitumour activity and low toxicity (10). Compound **C**, a 1,2,3-triazole-introduced oridonin derivative, showed strong anti-proliferative activity (IC_50_ = 1.94 μM), and its activity was about 3.52-fold than that of oridonin (IC_50_ = 6.84 μM) in HCT116 cancer cells (11).

**Figure 1:**
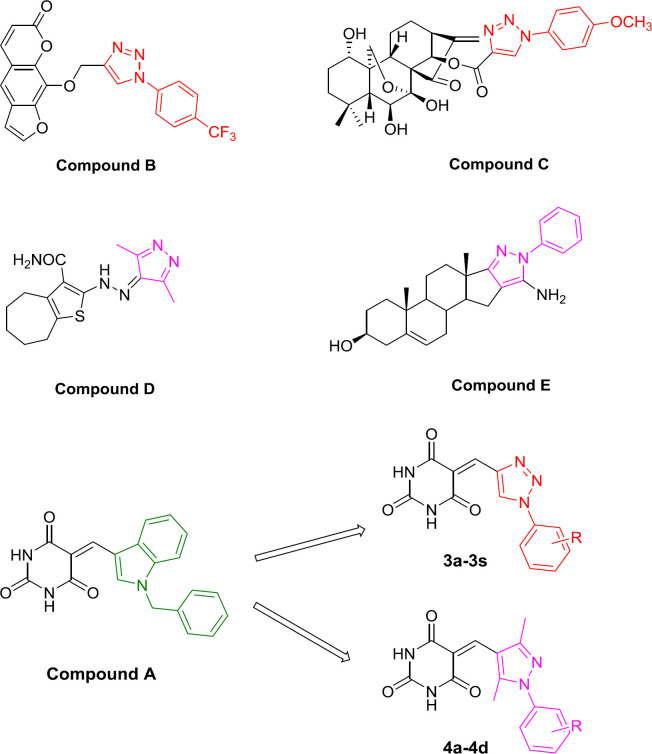
Design of target compound **3a-3s **and **4a-4d**

Pyrazoles also display a broad spectrum of biological activities, including antiproliferative anti-inflammatory effects (23). It was found that compound **D** with a pyrazole ring was a potent molecule against HCT116 and MCF-7 cell lines with IC_50_ values of 31.12 μM and 22.69 μM, respectively (22-24). The compound **E** exhibited potent anticancer activity and was less toxic for the human dermal fibroblast cells (Figure 1) (25). 

Recently, the molecular hybridization approach has been used as a drug design strategy and involves the combination of different pharmacophores to reach a novel compound in order to improve the efficacy compared to parent molecules. Due to the biological significance of indoles, combining molecular hybrids of indoles and barbituric acids is used as an anticancer agent. Singh *et al.* chose indole and barbituric acid entities to suitably combine them through the carbon-carbon bond formation to create new hybrid molecules (26). In the present study, we used other heterocycles 1-substituted phenyl-1,2,3-triazole and 1-substituted phenyl-3,5-methylpyrazole instead of indoles and designed, synthesized, and evaluated the antitumor biological activity of two series of new compounds (Figure 1).

## Experimental

*Materials and Methods*

*Synthesis*

Petroleum ether (PE), ethyl acetate (EA), ethanol (EtOH), *N*, *N*-Dimethylformamide (DMF), and other reagents were obtained commercially and were used without further purification. Solvents were dried according to standard procedures. Reactions were monitored by thin-layer chromatography (TLC) on silica gel plates. ^1^H-NMR and ^13^C-NMR spectra were measured on an AV-300 (Bruker, Flawil, Switzerland), and all chemical shifts were given in ppm relative to TMS. Mass spectra were measured on an HP1100LC (Agilent Technologies, Palo Alto, CA, USA). High-resolution mass spectra were measured on a MALDI-TOF/TOF mass spectrometer (Bruker Daltonik, Bremen, Germany).

*General procedure for synthesis of 5-((1-benzyl-1H-indol-3-yl)methylene)pyrimidine-2,4,6 (1H,3H,5H)-trione* ***(A)***

This compound was synthesized by following literature known methods (18).

Yellow solid, Yield: 69%. m.p 250 °C; ^1^H-NMR (300 MHz, DMSO-*d*_6_) δ: 11.13 (s, 1H, -CO-NH-), 11.05 (s, 1H, -CO-NH-), 9.61 (s, 1H, Indole-H), 8.68 (s, 1H,-CO-C=CH), 7.91 (t, *J *= 6 Hz, 1H, Ar-H), 7.69 (t, *J *= 6 Hz, 1H, Ar-H), 7.34 (t, *J *= 6 Hz, 7H, Ar-H), 5.67 (s, 2H, Ph-CH_2_-). ^13^C-NMR (75 MHz, DMSO-*d*_6_) δ: ^13^C-NMR (75 MHz, DMSO-*d*_6_) δ: 164.9, 163.6, 150.9, 143.4, 142.3, 137.0, 136.7, 130.3, 129.33, 128.5, 128.0, 124.3, 123.6, 118.4, 112.6, 111.2, 109.5, 50.8. ESI-HR MS (m/z): calcd. for C_20_H_16_N_3_O_3_^+^ [M + H]^+^: 346.1185; found: 346.1182.

*General Procedure for Preparation of* ***1a-1s***

Intermediate compounds 1a-1s are synthesized from aniline with different substitutions as raw materials through azide, click and oxidation reactions (27).

*General Procedure for Preparation of* ***2a-2d***

Intermediate compounds **2a-2d** are synthesized from phenylhydrazines with different substitutions as raw materials through the chemical reaction of cyclization and introduction of aldehyde groups (20).

*General Procedure for Preparation of 3a-3s and 4a-4d* (28)

**Scheme 1:**
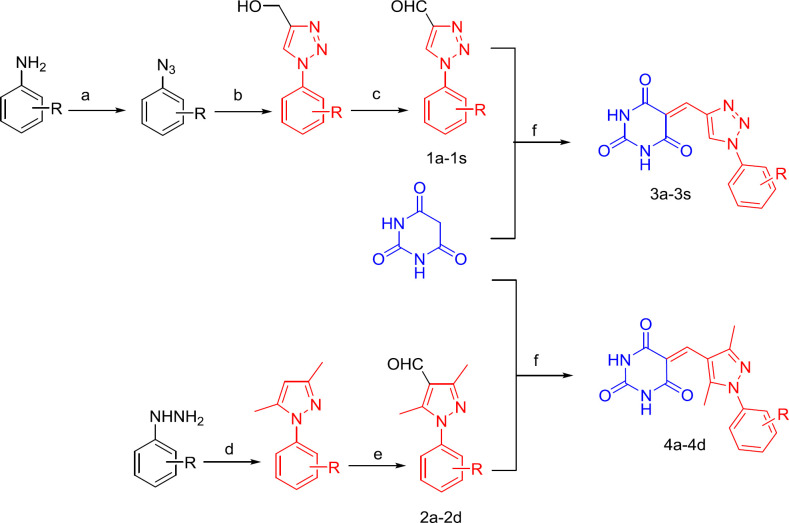
Reagents and conditions: (a) (i) NaNO_2_, HCl, 0  C, 30 min; (ii) NaN_3_, H_2_O, 0  C, 2-4 h; (b) propargyl alcohol, CuSO_4_·5H_2_O, sodium ascorbate, H_2_O:t-butanol=1:1, 24 h, rt; (c) MnO_2_/CH_3_COOC_2_H_5_, 1 h, reflux; (d) 2,4-Pentanedione, CH_3_CH_2_OH, reflux; (e) DMF, POCl_3_, 90  C; (f) H_2_O: CH_3_CH_2_OH=1:1(V:V), reflux, 45 min

A solution consisting of compound **1a-1s** and **2a-2d **(3 mmol) and Barbituric acid (3 mmol) in ethanol/water 1:1 (about 10 mL) at reflux for 45 min and cooled to room temperature. The solid was filtered off, rinsed twice with cold EtOH (15 mL each), dried in air, and recrystallized from EtOH (Scheme 1).

*5((1-phenyl-1H-1,2,3-triazol-4-yl)methylene)pyrimidine-2,4,6(1H,3H,5H)-trione (3a)*

White solid, Yield: 90%. m.p. 250 °C;^ 1^H-NMR (300 MHz, DMSO-*d*_6_) δ: 11.50 (s, 1H, -CO-NH-), 11.46 (s, 1H, -CO-NH-), 9.73 (s, 1H, triazole-H), 8.33 (s, 1H,-CO-C=CH), 7.96 (d, *J *= 6 Hz, 2H, Ar-H), 7.67-7.54 (m, 3H, Ar-H). ^13^C-NMR (75 MHz, DMSO-*d*_6_) δ: 163.3, 162.7, 150.6, 142.4, 141.3, 136.3, 130.5, 130.1, 129.8, 121.48, 117.6. ESI-HR MS (m/z): calcd. for C_13_H_10_N_5_O_3_^+^ [M + H]^+^: 284.0778; found: 284.0776.

*5-((1-(2-fluorophenyl)-1H-1,2,3-triazol-4-yl)methylene)pyrimidine-2,4,6(1H,3H,5H)-trione (3b)*

Yellow solid, Yield: 88%. m.p. 250 °C; ^1^H-NMR (300 MHz, DMSO-*d*_6_) δ: 11.50 (s, 1H, -CO-NH-), 11.45 (s, 1H, -CO-NH-), 9.74 (s, 1H, triazole-H), 8.35 (s, 1H,-CO-C=CH), 7.98 (t, *J *= 6 Hz, 1H, Ar-H), 7.71-7.59 (m, 2H, Ar-H), 7.49 (t, *J *= 6 Hz, 1H, Ar-H). ^13^C-NMR (75 MHz, DMSO-*d*_6_) δ: 163.3, 162.8, 154.2 (d, *J *= 249.75 Hz), 150.6, 142.1, 140.1, 132.90 (d, *J *= 6.0 Hz), 132.4 (d, *J *= 8.2 Hz), 126.21 (d, *J *= 3.7 Hz), 124.5 (d, *J *= 10.5 Hz), 118.0, 117.8, 117.6. ESI-HR MS (m/z): calcd. for C_13_H_9_FN_5_O_3_^+^ [M + H]^+^: 302.0684; found: 302.0678.

*5-((1-(3-fluorophenyl)-1H-1,2,3-triazol-4-yl)methylene)pyrimidine-2,4,6(1H,3H,5H)-trione (3c)*

White solid, Yield: 83%. m.p. 250 °C; ^1^H-NMR (300 MHz, DMSO-*d*_6_) δ: 11.51 (s, 1H, -CO-NH-), 11.47 (s, 1H, -CO-NH-), 9.73 (s, 1H, triazole-H), 8.29 (s, 1H,-CO-C=CH), 7.90-7.86 (m, 1H, Ar-H), 7.81 (d, *J *= 3 Hz, 1H, Ar-H), 7.71-7.63 (m, 1H, Ar-H), 7.44-7.38 (m, 1H, Ar-H). ^13^C-NMR (75 MHz, DMSO-*d*_6_) δ: 163.3, 162.6, 162.8 (d, *J *= 245.2 Hz), 150.6, 142.4, 141.1, 137.5 (d, *J *= 10.5 Hz), 130.0, 117.9, 117.3 (d, *J *= 45.0 Hz), 117.1 (d, *J *= 63 Hz), 109.3, 109.0. ESI-HR MS (m/z): calcd. for C_13_H_9_FN_5_O_3_^+^ [M + H]^+^: 302.0684; found: 302.0685.

*5-((1-(4-fluorophenyl)-1H-1,2,3-triazol-4-yl)methylene)pyrimidine-2,4,6(1H,3H,5H)-trione (3d)*

White solid, Yield: 75%. m.p. 250 °C; ^1^H-NMR (300 MHz, DMSO-*d*_6_) δ: 11.49 (s, 1H, -CO-NH-), 11.45 (s, 1H, -CO-NH-), 9.71 (s, 1H, triazole-H), 8.32 (s, 1H,-CO-C=CH), 8.01 (q, *J *= 3 Hz, 2H, Ar-H), 7.48 (t, *J *= 9 Hz, 2H, Ar-H). ^13^C-NMR (75 MHz, DMSO-*d*_6_) δ: 163.3, 162.7, 150.6, 142.4, 141.2, 133.0, 130.1, 124.2, 124.1, 117.7, 117.5, 117.2. ESI-HR MS (m/z): calcd. for C_13_H_9_FN_5_O_3_^+^ [M + H]^+^: 302.0684; found: 302.0679.

*5-((1-(2-chlorophenyl)-1H-1,2,3-triazol-4-yl)methylene)pyrimidine-2,4,6(1H,3H,5H)-trione (3e)*

White solid, Yield: 91%. m.p. 250 °C; ^1^H-NMR (300 MHz, DMSO-*d*_6_) δ: 11.49 (s, 1H, -CO-NH-), 11.43 (s, 1H, -CO-NH-), 9.68 (s, 1H, triazole-H), 8.36 (s, 1H,-CO-C=CH), 7.98 (t, *J *= 6 Hz, 1H, Ar-H), 7.83 (d, *J *= 3 Hz, 1H, Ar-H), 7.72-7.61 (m, 2H, Ar-H). ^13^C-NMR (75 MHz, DMSO-*d*_6_) δ: 163.3, 162.9, 150.6, 141.6, 141.0, 134.2, 133.9, 132.7, 131.1, 129.1, 128.9, 128.8, 118.0. ESI-HR MS (m/z): calcd. for C_13_H_9_ClN_5_O_3_^+^ [M + H]^+^: 318.0388; found: 318.0385.

*5-((1-(3-chlorophenyl)-1H-1,2,3-triazol-4-yl)methylene)pyrimidine-2,4,6(1H,3H,5H)-trione (3f)*

White solid, Yield: 73%. m.p. 250 °C;^1^H-NMR (300 MHz, DMSO-*d*_6_) δ: 11.50 (s, 1H, -CO-NH-), 11.45 (s, 1H, -CO-NH-), 9.71 (s, 1H, triazole-H), 8.26 (s, 1H,-CO-C=CH), 8.03 (d, *J *= 3 Hz, 1H, Ar-H), 7.91-7.88 (m, 1H, Ar-H), 7.66-7.57 (m, 2H, Ar-H). ^13^C-NMR (75 MHz, DMSO-*d*_6_) δ: 163.2, 162.6, 150.6, 142.4, 141.1, 137.3, 134.7, 132.1, 130.0, 129.8, 121.3, 120.2, 117.8. ESI-HR MS (m/z): calcd. for C_13_H_9_ClN_5_O_3_^+^ [M + H]^+^: 318.0388; found: 318.0385.

*5-((1-(4-chlorophenyl)-1H-1,2,3-triazol-4-yl)methylene)pyrimidine-2,4,6(1H,3H,5H)-trione (3g)*

White solid, Yield: 89%. m.p. 250 °C; ^1^H-NMR (300 MHz, DMSO-*d*_6_) δ: 11.50 (s, 1H, -CO-NH-), 11.46 (s, 1H, -CO-NH-), 9.74 (s, 1H, triazole-H), 8.32 (s, 1H,-CO-C=CH), 8.01 (d, *J *= 6 Hz, 2H, Ar-H), 7.70 (d, *J *= 3 Hz, 2H, Ar-H). ^13^C-NMR (75 MHz, DMSO-*d*_6_) δ: 163.3, 162.7, 150.6, 142.4, 141.1, 135.1, 134.4, 130.4, 129.8, 123.3, 117.8. ESI-HR MS (m/z): calcd. for C_13_H_9_ClN_5_O_3_^+^ [M + H]^+^: 318.0388; found: 318.0385.

*5-((1-(2,5-dichlorophenyl)-1H-1,2,3-triazol-4-yl)methylene)pyrimidine-2,4,6(1H,3H,5H)-trione (3h)*

White solid, Yield: 73%. m.p. 250 °C; ^1^H-NMR (300 MHz, DMSO-*d*_6_) δ: 11.50 (s, 1H, -CO-NH-), 11.44 (s, 1H, -CO-NH-), 9.71 (s, 1H, triazole-H), 8.34 (s, 1H,-CO-C=CH), 8.01 (d, *J *= 3 Hz, 1H, Ar-H), 7.83 (d, *J *= 3 Hz, 1H, Ar-H), 7.74 (dd, *J*_1_= 6 Hz, *J*_2_= 3 Hz, 1H, Ar-H). ^13^C-NMR (75 MHz, DMSO-*d*_6_) δ: 163.3, 162.8, 150.6, 141.6, 141.0, 135.0, 134.0, 133.0, 132.4, 128.7, 128.0, 118.0. ESI-HR MS (m/z): calcd. for C_13_H_8_Cl_2_N_5_O_3_^+^ [M + H]^+^: 351.9999; found: 351.9998.

*5-((1-(3,4-dichlorophenyl)-1H-1,2,3-triazol-4-yl)methylene)pyrimidine-2,4,6(1H,3H,5H)-trione (3i)*

Light yellow solid, Yield: 73%. m.p. 250 °C; ^1^H-NMR (300 MHz, DMSO-*d*_6_) δ: 11.50 (s, 1H, -CO-NH-), 11.44 (s, 1H, -CO-NH-), 9.71 (s, 1H, triazole-H), 8.37 (s, 1H,-CO-C=CH), 8.31 (s, 1H, Ar-H), 8.04-7.89 (m, 2H, Ar-H). ^13^C-NMR (75 MHz, DMSO-*d*_6_) δ: 163.2, 162.6, 150.6, 142.5, 140.9, 135.8, 132.9, 132.5, 132.2, 130.1, 123.4, 121.2, 118.0. ESI-HR MS (m/z): calcd. for C_13_H_8_Cl_2_N_5_O_3_^+^ [M + H]^+^: 351.9999; found: 351.9994.

*5-((1-(2-bromophenyl)-1H-1,2,3-triazol-4-yl)methylene)pyrimidine-2,4,6(1H,3H,5H)-trione (3j)*

Light yellow solid, Yield: 73%. m.p. 250 °C; ^1^H-NMR (300 MHz, DMSO-*d*_6_) δ: 11.49 (s, 1H, -CO-NH-), 11.43 (s, 1H, -CO-NH-), 9.64 (s, 1H, triazole-H), 8.37 (s, 1H,-CO-C=CH), 7.97 (d, *J *= 6 Hz, 1H, Ar-H), 7.79 (d, *J *= 3 Hz, 1H, Ar-H), 7.69-7.59 (m, 2H, Ar-H). ^13^C-NMR (75 MHz, DMSO-*d*_6_) δ: 163.3, 162.8, 150.6, 141.6, 141.0, 135.8, 134.2, 133.9, 133.0, 129.6, 129.2, 119.2, 117.9. ESI-HR MS (m/z): calcd. for C_13_H_9_BrN_5_O_3_^+^ [M + H]^+^: 361.9883; found: 361.9878.

*5-((1-(3-bromophenyl)-1H-1,2,3-triazol-4-yl)methylene)pyrimidine-2,4,6(1H,3H,5H)-trione (3k)*

White solid, Yield: 73%. m.p. 250 °C; ^1^H-NMR (300 MHz, DMSO-*d*_6_) δ: 11.50 (s, 1H, -CO-NH-), 11.47 (s, 1H, -CO-NH-), 9.79 (s, 1H, triazole-H), 8.33 (s, 1H,-CO-C=CH), 8.25 (s, 1H, Ar-H), 8.01 (d, *J *= 6 Hz, 1H, Ar-H), 7.79 (d, *J *= 3 Hz, 1H, Ar-H), 7.60 (T, *J *= 9 Hz, 1H, Ar-H). ^13^C-NMR (75 MHz, DMSO-*d*_6_) δ: 163.3, 162.6, 150.7, 145.4, 141.1, 137.5, 132.8, 132.3, 130.1, 124.2, 122.9, 120.7, 117.9. ESI-HR MS (m/z): calcd. for C_13_H_9_BrN_5_O_3_^+^ [M + H]^+^: 361.9883; found: 361.9884.

*5-((1-(4-bromophenyl)-1H-1,2,3-triazol-4-yl)methylene)pyrimidine-2,4,6(1H,3H,5H)-trione (3l)*

White solid, Yield: 96%. m.p. 250 °C; ^1^H-NMR (300 MHz, DMSO-*d*_6_) δ: 11.50 (s, 1H, -CO-NH-), 11.46 (s, 1H, -CO-NH-), 9.76 (s, 1H, triazole-H), 8.33 (s, 1H,-CO-C=CH), 7.96 (d, *J *= 6 Hz, 2H, Ar-H), 7.85 (d, *J *= 3 Hz, 2H, Ar-H). ^13^C-NMR (75 MHz, DMSO-*d*_6_) δ: 163.3, 162.7, 150.6, 142.5, 141.1, 135.6, 133.4, 129.8, 123.5, 122.9, 117.9. ESI-HR MS (m/z): calcd. for C_13_H_9_BrN_5_O_3_^+^ [M + H]^+^: 361.9883; found: 361.9878.

*5-((1-(2-methoxyphenyl)-1H-1,2,3-triazol-4-yl)methylene)pyrimidine-2,4,6(1H,3H,5H)-trione (3m)*

Yellow solid, Yield: 86%. m.p. 250 °C; ^1^H-NMR (300 MHz, DMSO-*d*_6_) δ: 11.47 (s, 1H, -CO-NH-), 11.41 (s, 1H, -CO-NH-), 9.66 (s, 1H, triazole-H), 8.35 (s, 1H,-CO-C=CH), 7.76 (d, *J *= 3 Hz, 1H, Ar-H), 7.59 (t, *J *= 9 Hz, 1H, Ar-H), 7.38 (d, *J *= 3 Hz, 1H, Ar-H), 7.19 (t, *J *= 9 Hz, 1H, Ar-H), 3.89 (s, 3H, -OCH_3_). ^13^C-NMR (75 MHz, DMSO-*d*_6_) δ: 163.4, 162.9, 151.9, 150.6, 141.6, 141.5, 133.6, 131.8, 126.1, 125.2, 121.6, 117.3, 113.7, 56.8. ESI-HR MS (m/z): calcd. for C_14_H_12_N_5_O_4_^+^ [M + H]^+^: 314.0884; found: 314.0879

*5-((1-(3-methoxyphenyl)-1H-1,2,3-triazol-4-yl)methylene)pyrimidine-2,4,6(1H,3H,5H)-trione (3n)*

Yellow solid, Yield: 93%. m.p. 250 °C; ^1^H-NMR (300 MHz, DMSO-*d*_6_) δ: 11.51 (s, 1H, -CO-NH-), 11.47 (s, 1H, -CO-NH-), 9.73 (s, 1H, triazole-H), 8.33 (s, 1H,-CO-C=CH), 7.56 (t, *J *= 6 Hz, 3H, Ar-H), 7.15 (d, *J *= 6 Hz, 1H, Ar-H), 3.88 (s, 3H, -OCH_3_). ^13^C-NMR (75 MHz, DMSO-*d*_6_) δ: 163.3, 162.7, 160.7, 150.6, 142.3, 141.3, 137.4, 131.4, 129.9, 117.7, 115.9, 113.6, 107.1, 56.2. ESI-HR MS (m/z): calcd. for C_14_H_12_N_5_O_4_^+^ [M + H]^+^: 314.0884; found: 314.0883.

*5-((1-(4-methoxyphenyl)-1H-1,2,3-triazol-4-yl)methylene)pyrimidine-2,4,6(1H,3H,5H)-trione (3o)*

White solid, Yield: 63%. m.p. 250 °C; ^1^H-NMR (300 MHz, DMSO-*d*_6_) δ: 11.48 (s, 1H, -CO-NH-), 11.44 (s, 1H, -CO-NH-), 9.63 (s, 1H, triazole-H), 8.32 (s, 1H,-CO-C=CH), 7.86 (t, *J *= 6 Hz, 2H, Ar-H), 7.17 (d, *J *= 6 Hz, 2H, Ar-H), 3.84 (s, 3H, -OCH_3_); ^13^C-NMR (75 MHz, DMSO-*d*_6_) δ: 163.7, 162.8, 160.4, 150.6, 142.2, 141.5, 129.7, 123.2, 117.3, 115.5, 56.1. ESI-HR MS (m/z): calcd. for C_14_H_12_N_5_O_4_^+^ [M + H]^+^: 314.0884; found: 314.0880.

*5-((1-(3,4-dimethoxyphenyl)-1H-1,2,3-triazol-4-yl)methylene)pyrimidine-2,4,6(1H,3H,5H)-trione (3p)*

Brown solid, Yield: 93%. m.p. 250 °C; ^1^H-NMR (300 MHz, DMSO-*d*_6_) δ: 11.50 (s, 1H, -CO-NH-), 11.45 (s, 1H, -CO-NH-), 9.67 (s, 1H, triazole-H), 8.33 (s, 1H,-CO-C=CH), 7.51-7.44 (m, 2H, Ar-H), 7.17 (d, *J *= 6 Hz, 1H, Ar-H), 3.88 (s, 3H, -OCH_3_), 3.85 (s, 3H, -OCH_3_). ^13^C-NMR (75 MHz, DMSO-*d*_6_) δ: 163.4, 162.7, 150.7, 150.1, 149.8, 142.2, 141.5, 130.0, 129.7, 117.4, 113.9, 112.5, 106.0, 56.4, 56.3. ESI-HR MS (m/z): calcd. for C_15_H_14_N_5_O_5_^+^ [M + H]^+^: 344.0989; found: 344.0985.

*5-((1-m-tolyl-1H-1,2,3-triazol-4-yl)methylene)pyrimidine-2,4,6(1H,3H,5H)-trione (3q)*

White solid, Yield: 93%. m.p. 250 °C; ^1^H-NMR (300 MHz, DMSO-*d*_6_)* δ*: 11.50 (s, 1H, -CO-NH-), 11.46 (s, 1H, -CO-NH-), 9.71 (s, 1H, triazole-H), 8.33 (s, 1H,-CO-C=CH), 7.76 (t, *J *= 9 Hz, 2H, Ar-H), 7.52 (t, *J *= 9 Hz, 1H, Ar-H), 7.39 (d, *J *= 3 Hz, 1H, Ar-H), 2.44 (s, 3H, -CH_3_). ^13^C-NMR (75 MHz, DMSO-*d*_6_) δ: 163.3, 162.7, 150.6, 142.3, 141.3, 140.4, 136.3, 130.6, 130.3, 129.6, 121.8, 118.5, 117.6, 21.3. ESI-HR MS (m/z): calcd. for C_14_H_12_N_5_O_3_^+^ [M + H]^+^: 298.0935; found: 298.0936.

*5-((1-p-tolyl-1H-1,2,3-triazol-4-yl)methylene)pyrimidine-2,4,6(1H,3H,5H)-trione (3r)*

White solid, Yield: 63%. m.p. 250 °C; ^1^H-NMR (300 MHz, DMSO-*d*_6_) δ: 11.50 (s, 1H, -CO-NH-), 11.46 (s, 1H, -CO-NH-), 9.68 (s, 1H, triazole-H), 8.32 (s, 1H,-CO-C=CH), 7.83 (d, *J *= 3 Hz, 2H, Ar-H), 7.44 (d, *J *= 3 Hz, 2H, Ar-H), 2.40 (s, 3H, -CH_3_). ^13^C-NMR (75 MHz, DMSO-*d*_6_) δ: 163.2, 162.8, 150.7, 142.3, 141.3, 139.9, 134.1, 130.9, 129.6, 121.4, 117.6, 21.2. ESI-HR MS (m/z): calcd. for C_14_H_12_N_5_O_3_^+^ [M + H]^+^: 298.0935; found: 298.0930.

*5-((1-(4-(trifluoromethyl)phenyl)-1H-1,2,3-triazol-4-yl)methylene)pyrimidine-2,4,6(1H,3H,5H)-trione (3s)*

White solid, Yield: 93%. m.p. 250 °C; ^1^H-NMR (300 MHz, DMSO-*d*_6_)* δ*: 11.52 (s, 1H, -CO-NH-), 11.48 (s, 1H, -CO-NH-), 9.86 (s, 1H, triazole-H), 8.33 (s, 1H,-CO-C=CH), 8.25 (d, *J *= 6 Hz, 2H, Ar-H), 8.03 (d, *J *= 6 Hz, 2H, Ar-H). ^13^C-NMR (75 MHz, DMSO-*d*_6_)* δ*: 163.3, 162.7, 150.7, 142.6, 140.8, 139.3, 130.1, 123.0 (d, *J *= 32.2 Hz), 127.8 (q, *J *= 3.7 Hz), 126.0, 122.3, 118.3. ESI-HR MS (m/z): calcd. for C_14_H_9_F_3_N_5_O_3_^+^ [M + H]^+^: 352.0652; found: 352.0657.

*5-((3,5-dimethyl-1-phenyl-1H-pyrazol-4-yl)methylene)pyrimidine-2,4,6(1H,3H,5H)-trione (4a)*

Yellow solid, Yield: 80%. m.p. 250 °C; ^1^H-NMR (300 MHz, DMSO-*d*_6_) δ: 11.29 (s, 1H, -CO-NH-), 11.15 (s, 1H, -CO-NH-), 8.20 (s, 1H,-CO-C=CH), 7.50 (s, 5H, Ar-H), 2.24 (s, 3H, -CH_3_), 2.22 (s, 3H, -CH_3_). ^13^C-NMR (75 MHz, DMSO-*d*_6_) δ: 163.7, 161.8, 151.3, 150.8, 145.7, 144.0, 138.9, 129.8, 128.7, 125.1, 116.7, 116.5, 13.9, 13.7. ESI-HR MS (m/z): calcd. for C_16_H_15_N_4_O_3_^+^ [M + H]^+^: 311.1133; found: 311.1133.

*5-((1-(3-fluorophenyl)-3,5-dimethyl-1H-pyrazol-4-yl)methylene)pyrimidine-2,4,6(1H,3H,5H)-trione (4b)*

Yellow solid, Yield: 85%. m.p. 250 °C; ^1^H-NMR (300 MHz, DMSO-*d*_6_) δ: 11.31 (s, 1H, -CO-NH-), 11.17 (s, 1H, -CO-NH-), 8.18 (s, 1H,-CO-C=CH), 7.60 (dd, *J*_1_= 15 Hz, *J*_2_= 9 Hz, 1H, Ar-H), 7.49-7.41 (m, 1H, Ar-H), 7.36-7.30 (m, 1H, Ar-H), 2.28 (s, 3H, -CH_3_), 2.21 (s, 3H, -CH_3_). ^13^C-NMR (75 MHz, DMSO-*d*_6_) δ: 163.3 (d, *J *= 70.5 Hz,), 161.3 (d, *J *= 66 Hz), 151.5, 150.8, 145.4, 144.1, 140.4 (d, *J *= 10.5 Hz), 131.4 (d, *J *= 9.75 Hz), 121.0 (d, *J *= 3.0 Hz), 117.4, 116.7, 115.4 (d, *J *= 21.0 Hz), 112.4, 112.1, 13.8, 13.7. ESI-HR MS (m/z): calcd. for C_16_H_14_FN_4_O_3_^+^ [M + H]^+^: 329.1039; found: 329.1038.

*5-((1-(3-chlorophenyl)-3,5-dimethyl-1H-pyrazol-4-yl)methylene)pyrimidine-2,4,6(1H,3H,5H)-trione (4c)*

Yellow solid, Yield: 93%. m.p. 250 °C; ^1^H-NMR (300 MHz, DMSO-*d*_6_) δ: 11.31 (s, 1H, -CO-NH-), 11.17 (s, 1H, -CO-NH-), 8.18 (s, 1H,-CO-C=CH), 7.65 (s, 1H, Ar-H), 7.61-7.51 (m, 3H, Ar-H), 2.27 (s, 3H, -CH_3_), 2.20 (s, 3H, -CH_3_). ^13^C-NMR (75 MHz, DMSO-*d*_6_) δ: 163.8, 161.8, 151.6, 150.8, 145.4, 144.1, 140.2, 134.1, 131.4, 128.5, 124.7, 123.5, 117.3, 116.8, 13.8, 13.7. ESI-HR MS (m/z): calcd. for C_16_H_14_ClN_4_O_3_^+^ [M + H]^+^: 345.0749; found: 345.0750.

*5-((3,5-dimethyl-1-p-tolyl-1H-pyrazol-4-yl)methylene)pyrimidine-2,4,6(1H,3H,5H)-trione (4d)*

Yellow solid, Yield: 85%. m.p. 250 °C; ^1^H-NMR (300 MHz, DMSO-*d*_6_) δ: 11.28 (s, 1H, -CO-NH-), 11.14 (s, 1H, -CO-NH-), 8.19 (s, 1H,-CO-C=CH), 7.38 (dd, *J*_1_= 21 Hz, *J*_2_= 6 Hz, 4H, Ar-H), 2.37 (s, 3H, Ar-CH_3_), 2.20 (s, 6H, -CH_3_). ^13^C-NMR (75 MHz, DMSO-*d*_6_) δ: 163.9, 161.8, 151.2, 150.8, 145.8, 144.0, 138.3, 136.5, 130.2, 124.9, 116.5, 116.3, 21.1, 13.8, 13.7. ESI-HR MS (m/z): calcd. for C_17_H_17_N_4_O_3_^+^ [M + H]^+^: 325.1290; found: 325.1292.

*Cell Cultures and Antiproliferative Assays* (11)

The antiproliferative activity of the target compounds against the panel of three different human cancer cell lines, liver (BEL-7402), colorectal (HCT-116), and breast (MCF-7) cell lines was evaluated using a standard MTT-based colorimetric assay. All cell lines were obtained from the Key Laboratory of Natural Resources and Functional Molecules of the Changbai Mountain (Yanbian University) and maintained in Dulbecco’s modified Eagle’s medium (DMEM) and RPMI Media 1640 (RPMI1640), supplemented with 10% foetal bovine serum (FBS) at 37 °C in a humidified atmosphere containing 5% CO_2_.

The cells were plated in 96-well plates at appropriate densities to ensure exponential growth throughout the experimental period (9 × 10^3^ cells per well) and then allowed to adhere for 24 h. the cells were then treated for 48 h with each compound. After 48 h of incubation, 10 μL of MTT solution was added to each well to a final concentration of 2 mg/mL. the plates were then incubated for a further 4 h. After incubation, the MTT solution was removed and 150 μL of DMSO was added to each well for coloration. The plates were shaken vigorously for 10 min at room temperature to ensure complete solubilisation. The optometric density (OD) was read on a microplate reader (EL × 800, BioTek, Highland Park, Winooski, VT, USA) at 492 nm, and the data were subsequently analysed. The percentage of cell growth inhibition was calculated from the following Equation:

Inhibitory rate (%) = [1 − (ODtreated − ODblank)/(ODcontrol − ODblank)] × 100%

To obtain the antiproliferative activity of compounds **3a-3s **and** 4a-4d**, the compounds were selected in the same way with four serial concentrations (1, 10, 50 and 100 μM) of those compounds. The optometric density (OD) reading was then used to calculate the IC_50_.

*Analysis for Apoptosis by Flow Cytometry* (29)

Apoptosis was detected using an Apoptosis Detection Kit (Invitrogen, Eugene, OR, USA). Briefly, BEL-7402 cells were cultured in 6-well plates (1.0 × 10^6^ cells per well) and incubated at 37 °C for 12 h. Cells with exponential growth were then incubated with compound **3s** at different concentrations (5 μM and 20 μM). Following 24 h of incubation, the cells were collected, washed twice with PBS and once with 1 × binding buffer, and then stained with 5 μM of annexin V-FITC and 2.5 μM of PI (5 mg/mL) in 1 × binding buffer for 30 min at 20 °C in the dark. Apoptotic cells were enumerated using a FACSCalibur flow cytometer with Cell Quest software (Becton–Dickinso, Franklin Lakes, NJ, USA).

## Results and Discussion

*Chemistry*

Compounds **1a–1s** were obtained in three synthetic steps, with overall yields ranging from 40% to 61%, based on procedures described previously (23). In the first step, the substituted aniline diazotization generated a substituted 1-azidobenzene, and in the second step, the substituted 1-azidobenzene with propargyl alcohol, CuSO_4_·5H_2_O, and *L*-ascorbic acid sodium salt generated a substituted phenyltriazole. The last step involved phenyltriazole oxidation by MnO_2_. Compounds **2a–2d** were obtained in two synthetic steps, with overall yields from 54% to 85%, based on the procedures described previously (20). In the first step, the substituted phenylhydrazine hydrochloride and 2,4-pentanedione in ethanol were refluxed for 4 h; in the second step, DMF and POCl_3_ underwent the Vilsmeier–Haack reaction. Compounds 3**a–3s** and 4**a–4d** were obtained by treating barbituric acid with the respective aldehydes (**1a–1s** and **2a–2d**) in 1:1 ethanol/water (about 10 mL) by refluxing for 45 min. Benzylidene barbiturates were obtained with yields of 63–96% (Scheme 1).

*In-vitro Antiproliferative Activity*

All synthesized compounds were evaluated for their antiproliferative activities *in vitro *against three human cancer cell lines (BEL-7402, MCF-7, and HCT-116) and compared with those of barbituric acid and 5-fluorouracil (5-FU). The cells were allowed to proliferate in the presence of the tested compounds for 48 h, and the results are presented as inhibition rates (Table 1) or half maximal inhibitory concentration (IC_50_) values (Table 2).

Some of the synthesized compounds showed highly significant antiproliferative effects. Among the compounds tested, compound **3s** exhibited the most potent activity against HCT-116 and MCF-7 cells with IC_50_ values of 21.53 µM and 8.98 µM, respectively. Compounds **3c**,** 3h** **3s** exhibited better bioactivities than 5-FU (*p* < 0.01) against BEL-7402 cells *in-vitro*. Compound **3h** exhibited potent activity against BEL-7402 and HCT-116 cells with IC_50_ values of 9.49 µM and 18.89 µM, respectively. Compound **3g** exhibited more better potent activity than 5-FU (*p* < 0.01) against HCT-116 cells with an IC_50_ value of 9.59 µM. 

*Structure-Activity Relationship Studies*

Based on an overall comparison, the compounds derived from structures with electron-withdrawing substituents on the 1,2,3-triazole ring exhibited potent activity, whereas those with electron-donating substituents on the 1,2,3-triazole ring displayed no apparent activity against the three cancer cell lines. For the 4-substituent compounds, only *4*-Cl and *4*-CF_3_ substitution compounds exhibited potent activity, and all 2-substituent compounds showed no significant activity. The special *2,5*-Cl_2_ replacement exhibited potent activity.

Against BEL-7402 cells, *4*-CF_3_ substitution exhibited potent activity, followed by *2,5*-Cl_2_, *3*-F, *3*-Cl, and *3*-Br; the compounds derived from structures with electron-withdrawing substituents on the 1,2,3-triazole ring exhibited potent activity. Against HCT-116 cells, *4*-Cl substitution exhibited potent activity, followed by *3*-Cl, *2,5*-Cl_2_, and *3*-Br; all the compound except *4*-Cl substitution displayed no apparent activity against HCT-116 cells. Against MCF-7 cells, only *4*-CF_3_ substitution exhibited potent activity.

Compound **3s**, which had a *4*-CF_3_ substitution, displayed the highest activity against BEL-7402 and MCF-7 cells; however, in HCT-116 cells, this compound exhibited lower activity. Compound **3g**, which had a *4*-Cl substitution, displayed activity only against HCT-116 cells, and for other cancer cells, this compound exhibited weaker activity.

*Selective Inhibition of Cancer Cell Growth by Compounds 3c, 3f, 3g, 3h, 3k, 3s and 5-FU*

The lack of selective cytotoxicity is the main factor that restricts the dose of most conventional chemotherapeutic agents (21). We compared the toxicities of compounds **3c, 3f, 3g, 3h, 3k **and** 3s** with 5-FU in L02 cells. Selectivity indexes between cancer cells and L02 cells were calculated. As shown in Table 3, compound **3s** exhibited 20.45-fold and 9.15-fold higher selectivities for BEL-7402 and MCF-7 cells than that for L02 cells, respectively. This selectivity displayed by compound **3s** was significantly higher than that displayed by 5-FU.

*Compound 3s Induces BEL-7402 Cell Apoptosis*

**Figure 2:**
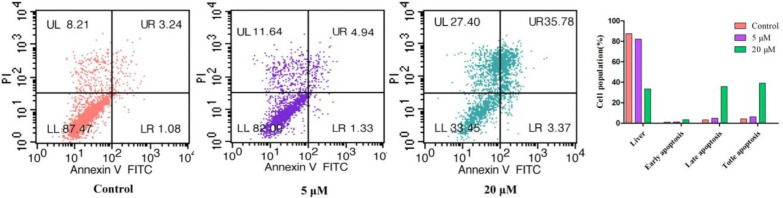
Apoptosis induction in BEL-7402 cancer cell after treatment of compound **3s** (5.0 µM, 20.0 µM, and no treatment (Ctrl) as a reference control for 24 h).

Numerous cytotoxic compounds exert their antiproliferative effect by apoptosis (22). Cell apoptosis analysis was performed to determine whether compound **3s** induced apoptosis of cells. As shown in Figure 2, the early-stage apoptosis rate of the control group was 1.08% and the late-stage apoptosis rate of the control group was 3.24%. The early-stage apoptosis rate gradually increased from 1.33% to 3.37% and the late-stage apoptosis rate increased from 4.94% to 35.78% after treatment with 5 μM and 20 μM of compound **3s**, respectively, for 24 h. However, we have found that the number of dead cells (UL) also increased from 8.21% (control) to 27.40% (20 µM). It is likely that compound 3s causes cell death by inducing apoptosis in combination with other ways. These results indicate that compound **3s** induced apoptosis and cell necrosis in a concentration-dependent manner and exert its anti-proliferative activity.

**Table 1:** The antiproliferative activity of compounds **3a-3s, 4a-4d **(Growth Inhibition at 100 μM).

**Compound**	**R**	**Growth Inhibition at 100 μM (%)**		
		BEL-7402	HCT-116	MCF-7
Barbituric acid	-	11.7 ± 1.0	14.3 ± 2.2	NA
A	-	19.6 ± 2.1	26.2 ± 1.8	11.8 ± 1.0
3a	4-H	54.0 ± 3.4	42.7 ± 2.5	30.9 ± 2.6
3b	2-F	34.1 ± 4.3	67.6 ± 3.8	27.5 ± 1.6
3c	3-F	79.0 ± 3.9	48.4 ± 2.3	34.6 ± 1.8
3d	4-F	31.9 ± 2.8	31.6 ± 1.9	34.1 ± 2.3
3e	2-Cl	39.6 ± 4.1	14.8 ± 0.9	19.8 ± 1.5
3f	3-Cl	75.2 ± 3.0	79.3 ± 5.6	58.0 ± 3.2
3g	4-Cl	46.6 ± 4.6	68.5 ± 4.6	23.9 ± 2.9
3h	2,5-Cl	82.1 ± 3.2	81.2 ± 4.2	92.2 ± 8.5
3i	3,4-Cl	47.2 ± 4.7	69.3 ± 2.9	14.2 ± 1.5
3j	2-Br	36.8 ± 2.1	46.3 ± 3.4	42.7 ± 2.6
3k	3-Br	81.6 ± 3.6	88.9 ± 3.1	31.7 ± 2.8
3l	4-Br	44.8 ± 3.8	49.8 ± 3.8	NA
3m	2-OCH_3_	25.6 ± 2.1	44.2 ± 3.4	7.9 ± 1.1
3n	3-OCH_3_	36.7 ± 19	67.1 ± 5.6	23.3 ± 3.5
3o	4-OCH_3_	28.4 ± 3.1	7.9 ± 0.6	22.6 ± 2.7
3p	3,4-OCH_3_	38.0 ± 1.9	43.9 ± 2.9	18.6 ± 1.7
3q	3-CH_3_	23.7 ± 2.1	94.8 ± 6.3	91.9 ± 2.4
3r	4-CH_3_	39.8 ± 3.1	52.5 ± 2.0	NA
3s	4-CF_3_	95.3 ± 4.9	62.9 ± 1.9	90.4 ± 5.3
4a	4-H	38.7 ± 1.0	30.9 ± 3.1	NA
4b	3-F	14.7 ± 1.2	9.1 ± 1.2	NA
4c	3-Cl	64.0 ± 2.1	58.7 ± 5.2	15.5 ± 1.1
4d	4-CH_3_	38.1 ± 1.3	28.4 ± 3.0	NA

NA: antiproliferative activity < 5%.

The values represent the means of three separate experiments.

**Table 2:** IC_50_ values (μM) of some active compounds

**Compounds**		**IC**_50_ ** values (μM)** ^a^		
**n**	**R**	**BEL-7402**	**HCT-116**	**MCF-7**
Barbituric acid	-	>100	>100	>100
3b	2-F	>100	78.19 ± 2.34	>100
3c	3-F	12.22 ± 0.91*	>100	>100
3f	3-Cl	15.94 ± 1.32	14.94 ± 0.55	29.14 ± 0.34
3g	4-Cl	>100	9.59 ± 1.01*	>100
3h	2,5-Cl	9.49 ± 1.78*	18.89 ± 1.97	75.92 ± 1.98
3i	3,4-Cl	>100	55.25 ± 2.31	>100
3k	3-Br	19.18 ± 1.80	19.39 ± 1.67	>100
3n	3-OCH_3_	>100	30.67 ± 2.10	>100
3q	3-CH_3_	>100	32.84 ± 1.90	29.79 ± 2.01
3s	4-CF_3_	4.02 ± 0.5*	21.53 ± 1.19	8.98 ± 0.99*
4c	3-Cl	48.18 ± 0.78	47.47 ± 3.21	>100
**5-FU**	-	21.30 ± 0.56	24.80 ± 0.78	28.11 ± 1.32

^a^IC_50_ : concentration that inhibits 50% of cell growth. The values represent the means of three separate experiments.

^*^p < 0.01 *vs.* 5-FU.

**Table 3:** *In-vitro* antiproliferative activities of compounds **3c, 3f, 3g, 3h, 3k, 3s** and 5-FU against normal cell line (L02).

**Compound**		**L02** **(IC**_50,_ **μM)** ^a^	**Selectivity index** ^b^		
**n**	**R**		**BEL-7402**	**HCT-116**	**MCF-7**
3c	3-F	82.33 ± 4.0	6.74	-^c^	-
3f	3-Cl	93.94 ± 5.9	5.89	6.28	3.22
3g	4-Cl	80.21 ± 6.0	-	8.36	-
3h	2,5-Cl	78.81 ± 3.9	8.30	4.17	1.04
3k	3-Br	66.42 ± 5.1	3.46	3.42	-
3s	4-CF_3_	82.23 ± 4.8	20.45	3.82	9.15
**5-FU**	-	19.12 ±1.0	0.90	0.65	0.68

^a^IC_50_: concentration that inhibits 50% of cell growth. The values represent the means of three separate experiments.

^b^SI: selective index (IC_50 _on normal cells/IC_50_ on tumor cells).

c-: Not calculated.

## Conclusion

In summary, we designed and synthesized two series of barbituric acid derivatives and evaluated their antiproliferative effects against three cancer cell lines. Several of the target compounds exhibited potent inhibitory activity *in-vitro*, and the antiproliferative activities of these compounds were screened via the MTT assay. In particular, compound **3s** exhibited excellent inhibitory activity against BEL-7402 cells, with an IC_50_ value of 4.02 µM. Moreover, it showed high inhibitory activities against MCF-7 cells, with IC_50_ values of 8.98 µM. Compound **3s** exhibited 20.45-fold and 9.15-fold higher selectivities for BEL-7402 and MCF-7 cells than that for L02 cells, respectively. Therefore, the modifications to the C-5 position of barbituric acid in the present study were helpful in improving its antiproliferative activity.

## Funding

 Project supported by the National Natural Science Foundation of China (No. 81960626) and the Doctor foundation of Yanbian University.
